# Bioactive Phenolics of *Lygeum spartum* L.: Insights Into Antioxidant, Anticholinesterase, and Antityrosinase Activities

**DOI:** 10.1002/cbdv.71652

**Published:** 2026-09-03

**Authors:** Zohra Arabi, Rania Arabi, Dilaycan Çam, Cansel Çakir, Mehmet Öztürk, Hamdi Bendif, Gharieb S. El‐Sayyad, Mohamed A. M. Ali, Walid Elfalleh, Stefania Garzoli

**Affiliations:** ^1^ Faculty of Nature and Life Sciences University Ibn Khaldoun Tiaret Algeria; ^2^ Laboratory of Agro‐Biotechnology and Nutrition in Semi‐arid Areas Faculty of Nature and Life Sciences University Ibn Khaldoun Tiaret Algeria; ^3^ Department of Chemistry Faculty of Sciences Muğla Sitki Kocman University Mugla Turkey; ^4^ Medical Laboratory Techniques Program Vocational School of Health Services Ankara Medipol University Ankara Turkey; ^5^ Department of Biology College of Science Imam Mohammad Ibn Saud Islamic University (IMSIU) Riyadh Saudi Arabia; ^6^ Department of Chemistry and Technologies of Drug Sapienza University Rome Italy

**Keywords:** antioxidant activity, enzyme inhibition, ferulic acid, HPLC–DAD, *Lygeum spartum* L., phenolic compounds

## Abstract

The phenolic composition of roots and leaves of *Lygeum spartum* L., a Mediterranean Poaceae species, was analyzed by HPLC–DAD, revealing an organ‐specific distribution of phenolic compounds, with ferulic acid identified as the predominant phenolic compound in the methanolic leaf extract (4.07 mg/g) and higher levels of flavonoids and phenolic acids in leaves than in roots. Antioxidant activity was evaluated using DPPH, ABTS^+^, and CUPRAC assays, while enzyme inhibitory activity was assessed against acetylcholinesterase (AChE), butyrylcholinesterase (BChE), and tyrosinase using L‐DOPA as the substrate. Methanolic extracts, particularly those from leaves, exhibited the highest antioxidant activity among the tested extracts (IC_50_ = 281.48 µg/mL for DPPH; 105.72 µg/mL for ABTS^+^; *A*_0_._5_ = 182.36 µg/mL for CUPRAC), whereas petroleum ether extracts were largely inactive. Enzyme inhibitory activity was generally weak, with all extracts exhibiting little or no inhibition of AChE and BChE, while the methanolic leaf extract showed weak tyrosinase inhibitory activity (IC_50_ = 310.69 µg/mL). To the best of our knowledge, this is the first study reporting the cholinesterase and tyrosinase inhibitory activities of *L. spartum* L. These results indicate that *L. spartum* is a source of phenolic compounds exhibiting moderate antioxidant activity, whereas its enzyme inhibitory effects are limited.

## Graphical abstract

Phenolic profile and bioactivity of *Lygeum spartum* L.

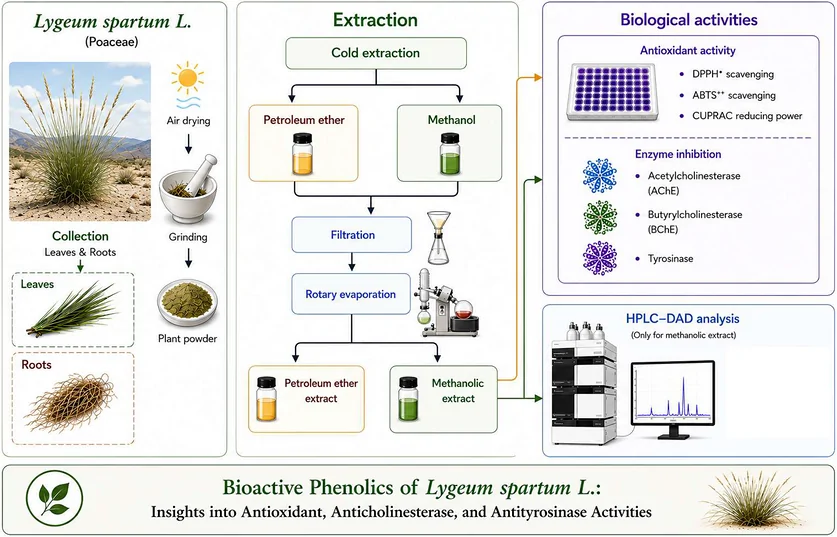

## 1 Introduction

Plants are recognized as one of the richest natural sources of structurally diverse bioactive compounds, including phenolic acids, flavonoids, tannins, alkaloids, terpenoids, and other specialized secondary metabolites that play essential roles in plant growth, defense, and adaptation to environmental changes and may provide diverse health‐related benefits in humans [1]. Recent studies have further highlighted the multifunctional biological potential of plant‐derived compounds. For instance, p‐coumaric acid has been shown to attenuate oxidative stress and inflammatory responses and to enhance antioxidant defense mechanisms, supporting its protective effects against chemically induced tissue damage [2]. Similarly, tannic acid has demonstrated antioxidative, anti‐inflammatory, and anti‐apoptotic properties in experimental models, further emphasizing the biological relevance of plant phenolic compounds [3]. Moreover, recent integrated investigations combining advanced phytochemical profiling with antioxidant and enzyme inhibition assays have demonstrated that plant extracts containing diverse secondary metabolites may exhibit multiple biological activities, highlighting the importance of linking chemical composition with biological effects [4]. These findings reinforce the view that plant‐derived secondary metabolites constitute a valuable reservoir of multifunctional compounds with potential applications in health‐related fields.

These specialized metabolites are synthesized through tightly regulated metabolic pathways in response to developmental and environmental cues and accumulate under abiotic and biotic stresses such as drought, salinity, ultraviolet radiation, extreme temperatures, and pathogen attack [5]. They protect plants by scavenging reactive oxygen species (ROS), chelating transition metal ions, regulating stress‐responsive pathways, and limiting oxidative damage [6, 7, 8]. Owing to these multifunctional properties, plant secondary metabolites have attracted considerable attention as natural antioxidants and enzyme inhibitors with promising applications in functional foods, nutraceuticals, pharmaceuticals, and cosmeceuticals [9, 10, 11].

Among plant secondary metabolites, phenolic compounds constitute one of the largest and most biologically important classes owing to their remarkable structural diversity and broad spectrum of biological activities [12]. They contribute to ultraviolet protection, cell wall reinforcement, lignification, and maintenance of cellular redox homeostasis, thereby enhancing plant tolerance to environmental stresses [10, 13]. Their biological effects are closely associated with their ability to regulate oxidative balance and cellular signaling. Phenolic compounds can directly scavenge ROS and reactive nitrogen species, modulate endogenous antioxidant defenses, and influence redox‐sensitive cellular pathways, thereby contributing to the maintenance of cellular homeostasis [14]. Recent studies have further demonstrated that individual plant polyphenols can act on molecular pathways relevant to human diseases. For example, integrative transcriptomic, network, and machine‐learning analyses identified potential molecular targets associated with genistein and resveratrol in Alzheimer's disease, highlighting the relevance of these polyphenols to mitochondrial and proteostasis‐related pathways [15]. Similarly, esculetin, a naturally occurring coumarin derivative, has been shown to attenuate doxorubicin‐induced renal injury by modulating processes involved in oxidative stress, inflammation, and apoptosis [16]. These findings illustrate the multifunctional nature of plant‐derived phenolic and related compounds and their potential to influence biological processes beyond direct radical scavenging. Beyond these antioxidant mechanisms, numerous phenolic acids and flavonoids have been reported to inhibit acetylcholinesterase (AChE), butyrylcholinesterase (BChE), and tyrosinase, enzymes involved in neurodegenerative disorders and abnormal melanogenesis [17, 18]. Consequently, plant phenolics are increasingly recognized as multifunctional natural compounds with potential applications in the prevention of oxidative stress‐related diseases, Alzheimer's disease, skin hyperpigmentation, and in the development of functional foods and natural therapeutic products [19, 20].

Although several Poaceae species have been investigated for their phytochemical composition and biological activities, many wild taxa remain insufficiently characterized [21, 22]. Members of this family produce diverse classes of secondary metabolites, including phenolic compounds, phenolic aldehydes, steroids, monoterpenes, and fatty acids, many of which exhibit antioxidant, antimicrobial, anti‐inflammatory, anticancer, and enzyme inhibitory properties, highlighting their therapeutic potential beyond their traditional agricultural importance [22]. Nevertheless, comprehensive studies simultaneously investigating phytochemical composition and biological activities in wild Poaceae species remain scarce, indicating that this family continues to represent an underexplored source of bioactive compounds.

Environmental conditions strongly influence the biosynthesis of plant secondary metabolites. Drought, one of the most severe abiotic stresses, disrupts cellular redox homeostasis through excessive ROS production, thereby activating the phenylpropanoid pathway and promoting the accumulation of phenolic compounds and other protective metabolites [23, 24]. Besides protecting plant tissues against oxidative damage, many of these stress‐induced metabolites exhibit antioxidant and enzyme inhibitory activities. Consequently, drought‐adapted species are increasingly regarded as valuable reservoirs of multifunctional phytochemicals with potential applications in the food, pharmaceutical, nutraceutical, and cosmetic industries.

*Lygeum spartum* L. is a perennial grass widely distributed throughout the arid and semi‐arid Mediterranean regions of North Africa, where it plays a key ecological role in stabilizing sandy soils and mitigating erosion and desertification. Owing to its strong and durable fibers, the species has traditionally been used as forage and in weaving [25]. Despite its ecological importance and remarkable adaptation to harsh environments, its phytochemical composition and biological properties remain poorly documented. Moreover, to the best of our knowledge, no previous studies have investigated the AChE, BChE, or tyrosinase inhibitory activities of this species, and the relationship between drought adaptation, phenolic accumulation, and these biological activities has not yet been elucidated.

Based on these considerations, we hypothesized that the adaptation of *L. spartum* to arid Mediterranean environments promotes the accumulation of phenolic compounds and other stress‐responsive secondary metabolites, which may contribute not only to its antioxidant capacity but also to its cholinesterase and tyrosinase inhibitory activities. Therefore, the present study aimed to provide a comprehensive phytochemical characterization of *L. spartum* roots (LS‐R) and leaves (LS‐L) using high‐performance liquid chromatography coupled with diode array detection (HPLC–DAD). The phenolic profile was investigated together with antioxidant activity using DPPH, ABTS•^+^, and CUPRAC assays, as well as inhibitory activity against AChE, BChE, and tyrosinase, to investigate whether the metabolite profile associated with adaptation to arid environments may contribute to the observed biological activities and support the potential of *L. spartum* as a source of natural bioactive compounds.

## 2 Materials and Methods

### 2.1 Materials

All solvents and reagents used in this study were of analytical grade. Petroleum ether, methanol (80%), ethanol, and acetic acid were purchased from Merck (Darmstadt, Germany). Reagents used for the antioxidant and enzyme inhibition assays, including DPPH, ABTS, neocuproine, CuCl_2_·2H_2_O, ammonium acetate (NH_4_Ac), kojic acid, L‐DOPA, DTNB, acetylthiocholine iodide, butyrylthiocholine chloride, and galantamine, were obtained from Sigma–Aldrich (St. Louis, MO, USA). AChE (AChE, EC 3.1.1.7) and BChE (BChE, EC 3.1.1.8) were also purchased from Sigma–Aldrich. Phenolic standards used for HPLC–DAD quantification were sourced from Sigma–Aldrich.

All chemicals and solvents were used without further purification.

The analytical instruments employed in this study included a SpectraMax 340PC384 microplate reader (Molecular Devices, USA), a Büchi R‐210 rotary evaporator (Büchi Labortechnik AG, Switzerland), and a Shimadzu 20AT HPLC–DAD system (Shimadzu, Japan).

### 2.2 Plant Material

**FIGURE 1:**
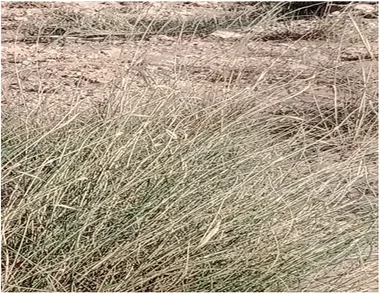
Photo of *L. spartum* L.

**FIGURE 2:**
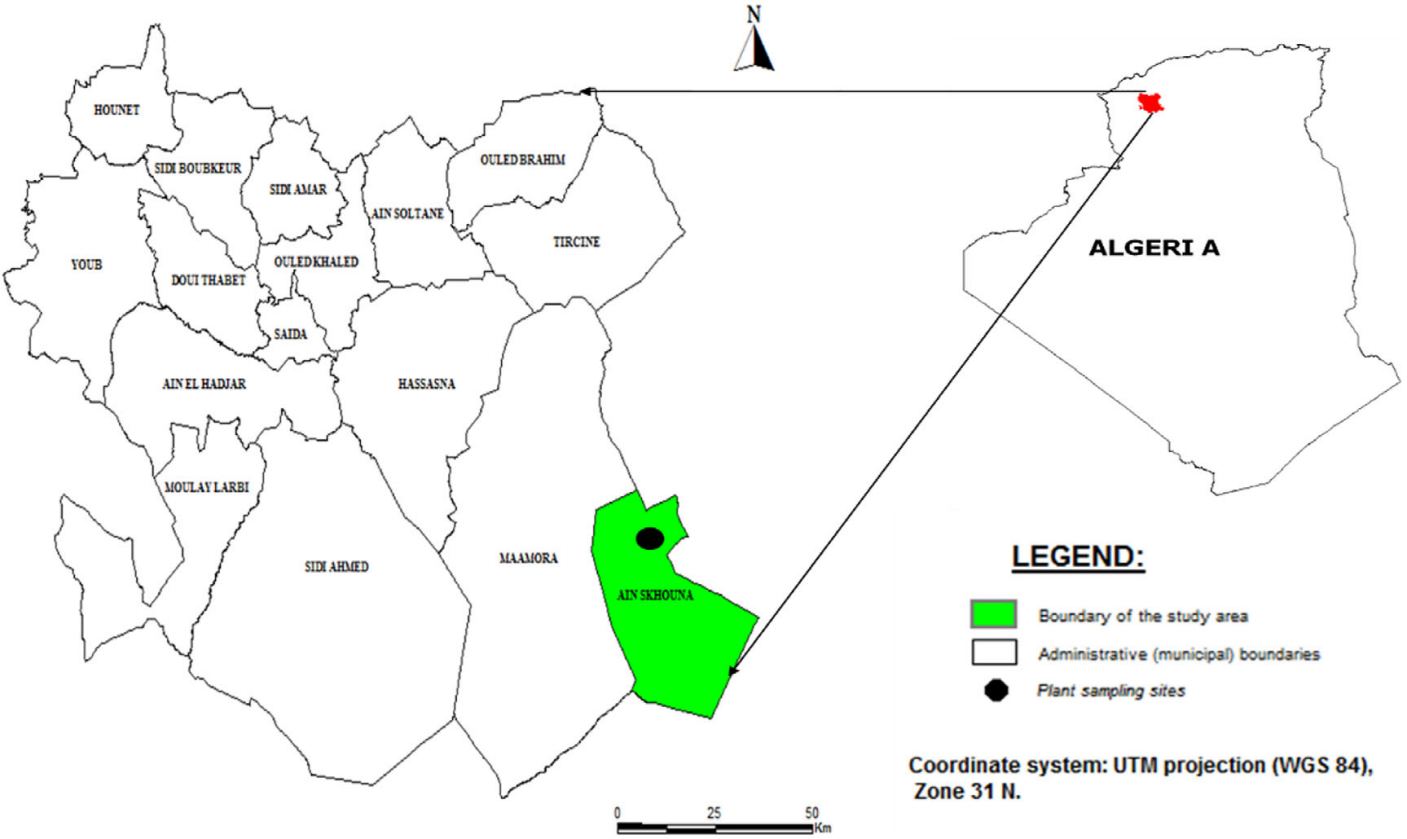
Study area localization map.

*L. spartum* L. was collected on May 21, 2022, in Ain Skhouan, located in the Saida region of Algeria, near Chott Chergui, an internationally important wetland (coordinates: 0°32′53.51″ E, 34°43′34.80″ N). Taxonomic identification of the plant material was carried out using the classical Flora of North Africa [26]. The taxonomic identification and nomenclature were confirmed by Dr. Zohra Arabi and Dr. Boubakr Saidi. A georeferenced observation of the collected specimen has been archived in iNaturalist (observation ID: 342788295) to provide publicly accessible documentation of the plant material used in this study. The plant material was air‐dried at ambient temperature, away from direct light. The roots were cut into small pieces, while the leaves were sieved and stored at 4°C until further use (Figures 1 and 2).

### 2.3 Extraction Process

The plant material was macerated separately in petroleum ether and 80% methanol. Each extraction was carried out in triplicate for 24 h at 30°C in the dark (Table 1). After filtration through Whatman No. 1 filter paper, the solvents were evaporated under reduced pressure using a Büchi R‐210 rotary evaporator. The final extracts were stored at 4°C until further use [27].

**TABLE 1:** Maceration conditions of the different parts of the plant.

Parts of the plant	Quantity (g)	Petroleum ether (mL)	Methanol (mL)
Roots	106	1400	1400
Leaves	151	900	900

### 2.4 HPLC–DAD Analysis

The chemical compositions of the methanolic extracts were determined using a reversed‐phase HPLC–DAD system following a validated method based on 27 reference standards [28]. A reversed‐phase Inertsil ODS‐3 column (150 mm × 4.0 mm i.d., 4 µm particle size) was used, and the column temperature was maintained at 35°C. The mobile phases consisted of 0.1% acetic acid in water (A) and methanol (B). The gradient elution program was performed over 40 min using the following conditions: 0–0.01 min (0%–20% B), 0.01–2 min (20%–60% B), 2–15 min (60%–80% B), 15–30 min (100% B), 30–35 min (100%–10% B), and 35–40 min (10%–0% B). The flow rate was 1 mL/min. Detection was performed using a diode array detector (DAD) at 254 nm, and the stock solutions were prepared according to Arab et al. [29].

### 2.5 Antioxidant Activity

#### 2.5.1 DPPH Free Radical Scavenging Assay

The free radical scavenging activity was evaluated using the DPPH assay originally described by Blois [30] and modified by Öztürk et al. [31]. All extracts, including those initially obtained using methanol or petroleum ether, were finally dissolved or diluted in ethanol to ensure complete solubility and compatibility with the DPPH solution. In a 96‐well microplate, 100 µL of extract solution at various concentrations were mixed with 100 µL of DPPH (0.1 mM in methanol). The mixtures were incubated for 1 h in the dark at room temperature, and the absorbance was measured at 517 nm using a SpectraMax microplate reader (Molecular Devices, CA, USA). Ethanol, as the final solvent of all samples, was used as the negative control (vehicle), and BHA served as the positive antioxidant standard [32].

The percentage of DPPH radical scavenging activity was calculated according to the following equation:

$$\text{DPPH radical scavenging activity}\;\left(\%\right)\;=\;\left[\frac{A_{\text{control}}-A_{\text{sample}}}{A_{\text{control}}}\right]\times 100$$

where $A_{\mathrm{control}}$ is the absorbance of the control and $A_{\mathrm{sample}}$ is the absorbance of the reaction mixture containing the extract. The radical scavenging capacity was expressed as IC_50_ values (µg/mL), representing the concentration of the extract required to scavenge 50% of the DPPH radicals.

#### 2.5.2 ABTS Radical Cation Decolorization Assay

The ABTS^+^ radical scavenging activity was measured following the method of Re et al. [33], with minor modifications described by Öztürk et al. [31]. ABTS•^+^ was generated by reacting 7 mM ABTS with 2.45 mM potassium persulfate and allowing the mixture to stand in the dark for 12–16 h. The radical solution was then diluted with distilled water to obtain an absorbance of 0.70 ± 0.01 at 734 nm. In a 96‐well microplate, 160 µL of ABTS•^+^ solution was mixed with 40 µL of extract solution (dissolved in ethanol). After incubation for 30 min at room temperature, the absorbance was measured at 734 nm. Ethanol was used as the negative control, and BHA was used as the positive control [32].

The percentage of ABTS•^+^ radical scavenging activity was calculated according to the following equation:

$$\begin{array}{ccc} & & {\text{ABTS}}^{+}\;\text{radical}\;\;\text{scavenging}\;\;\text{activity}\left(\%\right)\\ & & =[\frac{(A_{control}-A_{sample})}{A_{control}}]\times 100 \end{array}$$

where $A_{\mathrm{control}}$ is the absorbance of the control and $A_{\mathrm{sample}}$ is the absorbance of the reaction mixture containing the extract. The radical scavenging capacity was expressed as IC_50_ values (µg/mL), representing the concentration of the extract required to scavenge 50% of the ABTS•^+^ radicals.

#### 2.5.3 Cupric Reducing Antioxidant Capacity

The cupric‐reducing antioxidant capacity (CUPRAC) was determined according to Apak et al. [34], with modifications described by Öztürk et al. [31]. In a 96‐well microplate, 60 µL of ammonium acetate buffer (1 M, pH 7.0) was mixed with 50 µL of neocuproine (7.5 mM) and 50 µL of CuCl_2_·2H_2_O (10 mM), followed by 40 µL of extract solution (dissolved in ethanol) at various concentrations. The mixture was incubated for 1 h at room temperature, and the absorbance was measured at 450 nm using a SpectraMax microplate reader. BHA was used as the positive antioxidant standard [32]. The reducing antioxidant capacity was expressed as *A*_0_._5_ values (µg/mL), defined as the concentration of the extract required to produce an absorbance of 0.500 at 450 nm.

### 2.6 Anticholinesterase Activity

AChE and BChE inhibitory activities were measured by the Ellman method [35] using a 96‐well plate microplate reader. All conditions were described in [31]. Briefly. 20 µL of AChE enzyme (5.32 × 10^−3^ U) or BChE (6.85 × 10^−3^ U) was incubated for 15 min at 25°C with 150 µL of buffer with 0.1 M sodium phosphate (pH 8.0) and 10 µL of sample at different concentrations. The reaction was then started by adding 10 µL of iodide acetylthiocholine (7.1 × 10^−4^ M) or butyrylthiocholine chloride (2 × 10^−4^ M) and 10 µL of DTNB (5 × 10^−4^ M). The absorbance of the yellow 5‐thio‐2‐nitrobenzoate anion produced is measured at 412 nm every five minutes for 15 min. The positive control used was galantamine at the same concentrations as the samples. The percentage inhibition of AChE and BChE is determined relative to the blank (methanol with phosphate buffer pH = 8) by the formula [(Enzyme activity without extract—Enzyme activity with the extract)/ Enzyme activity without extract] × 100. Galantamine was used as a reference compound. The results were given as percentage inhibition against 25, 50, 100, and 200 µg/mL concentrations.

### 2.7 Tyrosinase Inhibitory Activity

Tyrosinase inhibition (EC 1.14.18.1, 250 KU, mushroom tyrosinase, Sigma) was assessed using the modified DOPAchrome method with L‐DOPA as the substrate [36, 37]. Tyrosinase is a copper‐containing oxidase that catalyzes the hydroxylation of L‐tyrosine to L‐DOPA (monophenolase activity) and the subsequent oxidation of L‐DOPA to DOPAchrome (diphenolase activity), a key intermediate in melanin biosynthesis. Therefore, monitoring the formation of DOPAchrome at 475 nm provides a reliable and widely used approach for evaluating tyrosinase inhibitory activity [38].

In brief, 150 µL of 50 mM sodium phosphate buffer (pH 6.8), 10 µL of the sample in ethanol (or the control), and 20 µL of tyrosinase enzyme solution were pipetted into a 96‐well microplate and incubated at 37°C for 10 min. After incubation, 20 µL of 0.5 mM L‐DOPA was added to initiate the reaction, and the enzyme activity was monitored by measuring the absorbance at 475 nm over 10 min to follow the formation of DOPAchrome. A reduction in the rate of DOPAchrome formation relative to the control was considered indicative of tyrosinase inhibition by the tested extracts [38].

Tyrosinase inhibition was calculated by comparing the reaction rates of the samples with the blank (ethanol in phosphate buffer, pH 6.8) using the following formula:

$$\mathrm{Inhibition}\;\%\;=\frac{E-S}{E}\times 100$$

where *E* represents the enzyme activity without the test sample and *S* represents the enzyme activity with the test sample. Kojic acid was used as the positive control.

### 2.8 Statistical Analysis

All biological activity assays were performed using triplicate technical measurements for each extract concentration. The results were expressed as mean ± standard error of the mean (SEM). IC_50_ and *A*_0_._5_ values were calculated from concentration–response curves generated using serial dilutions of the extracts.

## 3 Results and Discussion

### 3.1 HPLC–DAD Analysis

The phenolic profile of *L. spartum* L. extracts was characterized by HPLC–DAD, and the results are summarized in Table 2.

**TABLE 2:** Phenolic composition of *L. spartum* L. extracts determined by HPLC–DAD (mg/g dry extract), and retention times, calibration equations, regression coefficients (*R* ^2^), linearity ranges, LODs, LOQs, recoveries, and within‐day precision of phenolic standards.

Phenolic compounds	RT^a^ (min)	LS‐R	LS‐L	Calibration Equation	R^2^ ^b^	Linear Range (µg/mL)	Detection wavelength	LOD^c^ (µg/mL)	LOQ^c^ (µg/mL)	Recovery (%)	RSD ^d^ within day (n = 7)
Theobromine	25.97	*tr*	*tr*	*y* = 3942.7x + 81451	0.9993	5.00–1200	254	3.75	11.36	94.02	2.51
Theophylline	29.45	*tr*	*tr*	*y* = 36694x + 68674	0.9998	1.00–450	254	0.67	2.03	96.88	2.89
Catechin	30.27	*tr*	1.03	*y* = 2611.2x + 74392	0.9972	3.80–1950	254	1.20	3.63	96.14	3.78
p‐Hydroxybenzaldehyde	33.37	*—*	0.06	*y* = 34376x + 4239.6	0.9880	1.25–50	280	1.33	4.44	99.01	4.76
6,7‐Dihydroxycoumarin	33.44	*tr*	*tr*	*y* = 27607x + 208788	0.9963	1.00–60	254	1.02	3.08	98.16	3.88
Methyl‐p‐benzoquinone	34.00	*tr*	1.03	*y* = 37060x − 637474	0.9934	6.05–387.5	254	1.58	4.78	103.37	4.86
Vanillic acid	34.76	*—*	0.06	*y* = 66764x + 46508	0.9998	0.39–200	254	0.29	0.88	100.16	5.06
Epicatechin	35.28	0.59	0.14	*y* = 2097.6x + 7998.2	0.9999	3.9–500	254	0.95	2.86	101.25	4.00
Chlorogenic acid	39.60	0.18	0.28	*y* = 46920x − 36953	0.9993	2.00–180	254	1.56	4.74	98.62	4.74
Ferulic acid	42.56	*tr*	4.07	*y* = 44908x + 29138	0.9992	0.80–102	254	0.39	1.18	95.19	3.21
Cynarin	44.50	0.88	0.88	*y* = 42559x − 2E+06	0.9959	32.0–512	254	24.52	74.30	94.91	3.52
Coumarin	45.18	*tr*	*tr*	*y* = 95160x − 34748	0.9997	1.42–45.5	254	1.02	3.09	96.30	3.59
Hesperidin	47.38	0.48	2.68	*y* = 2949.5x + 9083.5	0.9995	3.00–180	254	2.99	9.73	97.28	2.68
*trans*‐2‐Hydroxycinnamic acid	48.24	0.09	0.29	*y* = 53442x + 104662	0.9996	0.35–355	254	0.38	1.16	94.71	2.85
Genistein	57.74	0.03	0.10	*y* = 69160x + 5753.8	0.9996	4.74–615	254	2.20	10.14	94.49	3.11
Apigenin	64.07	0.32	1.11	*y* = 71990x + 62472	0.9996	0.66–170	254	0.65	1.96	98.80	1.36
Curcumin	72.62	0.39	*—*	*y* = 15850x + 140009	0.9932	1.56–100	254	1.11	3.36	95.63	2.17

*Note*: —: not detected, *tr*: trace amount.

^a^ RT: retention time of the compound in minutes.

^b^ *R* ^2^: linearity of the calibration graph.

^c^ LOD: limit of Detection in µg/mLand LOQ: limit of Quantification in µg/mL.

^d^ RSD: percentage relative standard deviation.

**FIGURE 3:**
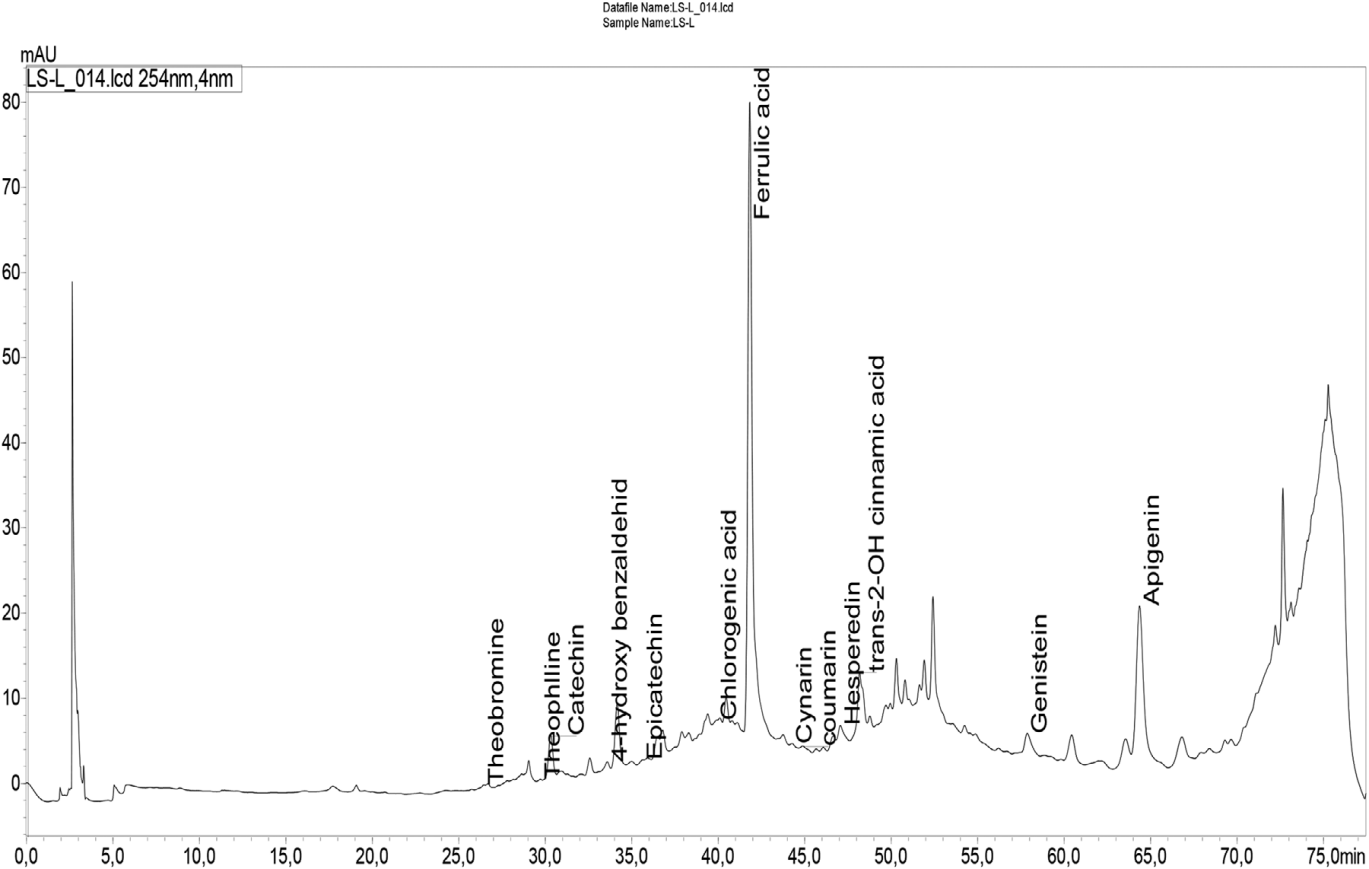
HPLC–DAD chromatogram of LS‐L‐MeOH at 254 nm (Inertsil ODS‐3 column 150 mm × 4.0 mm i.d. 4 µm film thickness). Mobile phase 0.1% acetic acid‐methanol (gradient elution). Flow rate 1 mL/min. Diode array detection 254 nm.).

**FIGURE 4:**
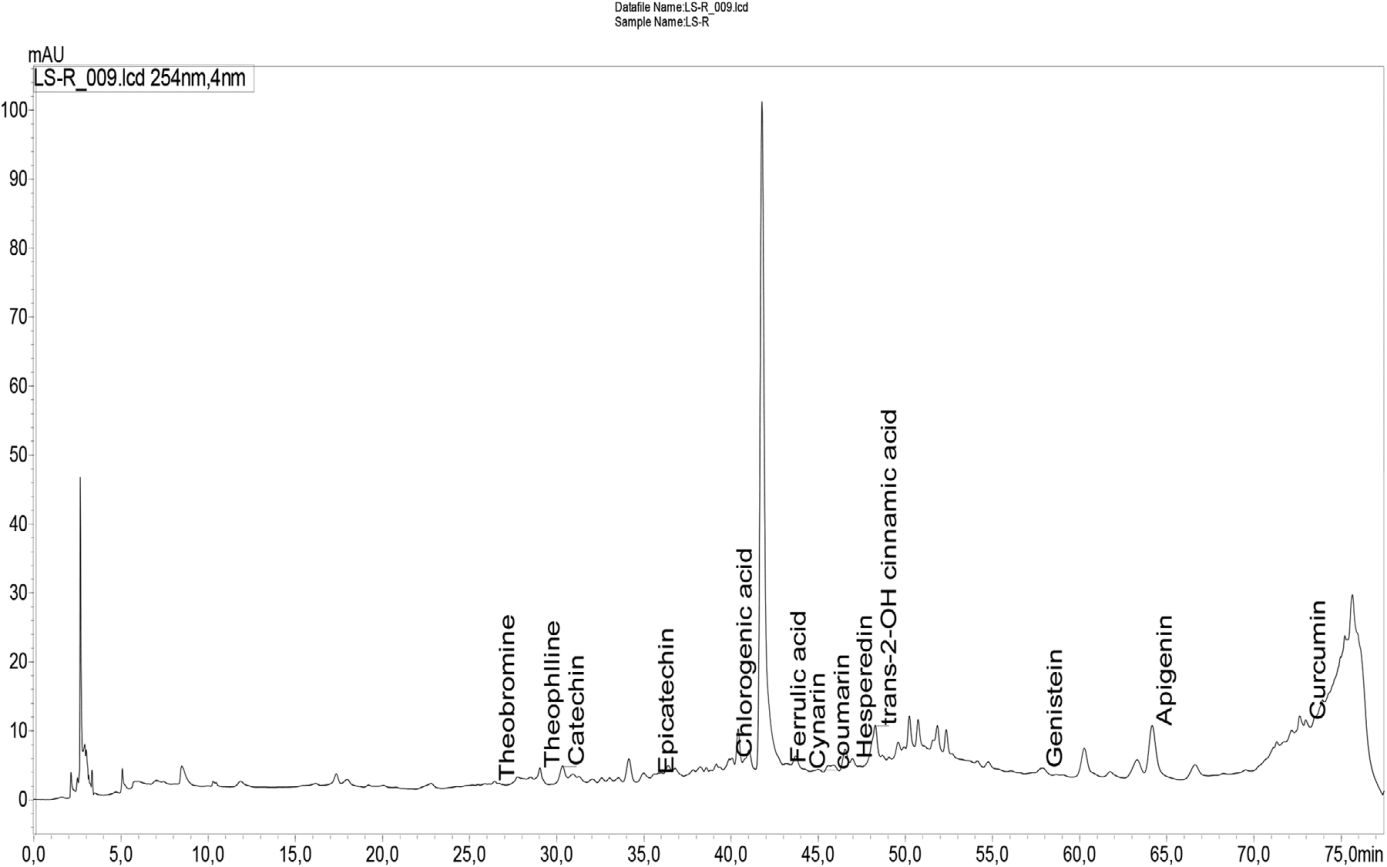
HPLC–DAD chromatogram of LS‐R‐MeOH at 254 nm (Inertsil ODS‐3 column 150 mm × 4.0 mm i.d. 4 µm film thickness). Mobile phase 0.1% acetic acid‐methanol (gradient elution). Flow rate 1 mL/min. Diode array detection 254 nm.).

The phenolic composition of *L. spartum* L., analyzed by HPLC–DAD (Table 2; Figures 3 and 4), revealed a diverse profile of bioactive compounds with an organ‐specific distribution. Ferulic acid was identified as the predominant phenolic compound in the leaf extract (LS‐L) 4.07 mg/g. Several other compounds, including theobromine, theophylline, 6,7‐dihydroxycoumarin, and coumarin, were detected only in trace amounts. A clear organ‐dependent pattern was observed: leaves accumulated higher concentrations of flavonoids and phenolic acids such as catechin (1.03 mg/g), hesperidin (2.68 mg/g), apigenin (1.11 mg/g), and ferulic acid (4.07 mg/g), whereas roots contained comparatively lower concentrations of phenolic compounds, with cynarin (0.88 mg/g), epicatechin (0.59 mg/g), curcumin (0.39 mg/g), and apigenin (0.32 mg/g) among the most abundant detected metabolites. Some metabolites were exclusively detected in one organ, reflecting tissue‐specific metabolic regulation: catechin and methyl‐*p*‐benzoquinone were found only in leaves, while curcumin was confined to roots. These observations indicate that leaves are the main site for flavonoid accumulation, likely contributing to photoprotection and antioxidant defense, whereas roots may preferentially accumulate metabolites involved in soil interaction, stress tolerance, and defense.

The predominance of ferulic acid in the leaf extract is particularly noteworthy because this hydroxycinnamic acid is one of the most abundant phenolic acids in plants, especially within the Poaceae family, where it plays important structural and physiological roles by contributing to cell wall organization, lignification, and adaptation to environmental stresses [39]. In addition, numerous studies have demonstrated that isolated ferulic acid possesses antioxidant, anti‐inflammatory, antimicrobial, and cytoprotective properties [40, 41]. These biological properties have been reported for isolated compounds or other plant species and are presented here primarily to provide phytochemical context for the major constituents identified in *L. spartum*.

Overall, the HPLC–DAD analysis highlights the richness and organ‐dependent distribution of phenolic compounds in *L. spartum*, with ferulic acid and several flavonoids representing the principal constituents of the leaf extract. These findings expand the current phytochemical knowledge of this species and highlight its phytochemical potential as a natural source of phenolic compounds. Further phytochemical investigations, particularly bioassay‐guided fractionation and the isolation of individual constituents, would contribute to a better understanding of the chemical diversity and biological relevance of the major metabolites identified in *L. spartum*.

### 3.2 Antioxidant Activity

The antioxidant activity of *L. spartum* L. extracts was evaluated using the DPPH, ABTS, and CUPRAC assays. The results are summarized in Table 3, which presents the IC_50_ values (µg/mL) for the DPPH and ABTS assays and the *A*_0_._5_ values (µg/mL) for the CUPRAC assay.

**TABLE 3:** Antioxidant activity of *L. spartum* L. extracts determined by the DPPH, ABTS^+^, and CUPRAC assays.

Samples	Extract	Antioxidant activity		
		DPPH free radical scavenging assay	ABTS free radical scavenging assay	Cupric reducing antioxidant capacity
		IC_50_ (µg/mL)	IC_50_ (µg/mL)	*A*_0_._5_ (µg/mL)
Roots	PE MeOH	*NA* 365.36 ± 11,85	*>800* 109.90 ± 1.67	435.2 ± 6.67 212.67 ± 7.34
Leaves	PE MeOH	*NA* 281.48 ± 11.10	*NA* 105.72 ± 2.53	607.5 ± 25.24 182.36 ± 7.31
BHA^a^		4.23 ± 0.08	2.05 ± 0.04	5.30 ± 0.06

^a^ butylated hydroxyanisole.

The antioxidant activity of *L. spartum* L. extracts was evaluated using the DPPH, ABTS^+^, and CUPRAC assays, revealing a clear influence of both the extraction solvent and the plant organ. Overall, methanolic extracts exhibited higher antioxidant activity than petroleum ether extracts, confirming the greater efficiency of polar solvents for extracting phenolic compounds associated with radical‐scavenging activity.

Among the methanolic extracts, the leaf extract (LS L MeOH) exhibited the highest antioxidant activity, with IC_50_ values of 281.48 ± 11.10 µg/mL for DPPH, 105.72 ± 2.53 µg/mL for ABTS^+^, and *A*_0_._5_ = 182.36 ± 7.31 µg/mL for the CUPRAC assay. The methanolic root extract (LS R MeOH) showed slightly lower antioxidant activity, with IC_50_ values of 365.36 ± 11.85 µg/mL for DPPH, 109.90 ± 1.67 µg/mL for ABTS^+^, and *A*_0_._5_ = 212.67 ± 7.34 µg/mL for the CUPRAC assay. In contrast, the petroleum ether extracts (LS L PE and LS R PE) exhibited negligible antioxidant activity, which may be attributed to the limited extraction of phenolic compounds by the non‐polar solvent.

HPLC–DAD analysis revealed that the relatively higher antioxidant activity of the methanolic leaf extract (LS L MeOH) may be associated with its higher content of bioactive phenolics, particularly ferulic acid, hesperidin, catechin, apigenin, and chlorogenic acid. Ferulic acid was identified as the predominant phenolic compound in the methanolic leaf extract (LS L MeOH) (4.07 mg/g), whereas hesperidin (2.68 mg/g), apigenin (1.11 mg/g), catechin (1.03 mg/g), and chlorogenic acid (0.28 mg/g) were also present at relatively higher concentrations than in the root extract. These phenolic compounds have previously been reported to possess antioxidant properties and may contribute to the radical‐scavenging activity observed in the methanolic extract. However, their individual contributions cannot be established from the present study.

Ferulic acid, the predominant phenolic compound identified in the methanolic leaf extract (LS L MeOH), has been widely reported to possess antioxidant activity through free‐radical scavenging and other antioxidant mechanisms [41, 42]. These antioxidant properties have been demonstrated for isolated ferulic acid or extracts from other plant species under different experimental conditions and are discussed here solely to provide phytochemical context for the compounds identified in *L. spartum*. Therefore, the present study does not allow the antioxidant activity observed in the methanolic leaf extract (LS L MeOH) to be attributed specifically to ferulic acid.

Although the antioxidant activity of the *L. spartum* extracts was considerably lower than that of the synthetic antioxidant BHA (DPPH IC_50_ = 4.23 µg/mL), the methanolic leaf extract exhibited the highest antioxidant activity among the tested plant extracts. This observation suggests a relationship between phenolic composition and radical‐scavenging activity. Therefore, the methanolic extracts of *L. spartum* may represent a potential natural source of bioactive phenolic compounds exhibiting moderate antioxidant activity and deserve further investigation regarding their possible pharmaceutical, nutraceutical, and food applications.

To contextualize the antioxidant activity of *L. spartum* L. within the Poaceae family, comparative data from previous studies on grasses provide valuable reference points. In the study by Aliyu et al. [43], methanolic extracts from ten Poaceae species were evaluated for DPPH radical‐scavenging activity. The results showed a wide spectrum of antioxidant capacities, with *Panicum maximum* exhibiting the strongest activity (IC_50_ = 4.73 µg/mL), followed by *Eragrostis ciliaris* (16.49 µg/mL) and *Leersia hexandra* (17.78 µg/mL). Moderate activity was observed for *Imperata cylindrica* (35.87 µg/mL), *Cynodon dactylon* (42.89 µg/mL), *Dactyloctenium aegyptium* (80.15 µg/mL), and *Eleusine indica* (92.95 µg/mL). The species with the weakest activity included *Andropogon gayanus* (99.89 µg/mL), *Pennisetum purpureum* (112.07 µg/mL), and *Urelytrum muricatum* (137.22 µg/mL).

In addition, Gebashe et al. [22] evaluated the antioxidant activity of methanolic extracts from thirteen medicinal grasses (also Poaceae), reporting DPPH EC_50_ values ranging from 0.02 to 0.11 mg/mL (20–110 µg/mL). These findings indicate that many grasses possess high radical‐scavenging potential when rich in phenolic compounds.

When compared to these references, the methanolic leaf extract of *L. spartum* L. (LS L MeOH, IC_50_ = 281.48 µg/mL) and root extract (LS R MeOH, IC_50_ = 365.36 µg/mL) exhibited moderate antioxidant activity. Although their radical‐scavenging strength was lower than that of the most active Poaceae species, the richness in bioactive phenolics, especially ferulic acid may contribute to the observed antioxidant activity and supports the biological potential of *L. spartum*.

In conclusion, the results highlight that methanolic extracts of *L. spartum* L., particularly from leaves, represent a potential source of bioactive phenolic compounds exhibiting moderate antioxidant activity. The study underscores the importance of solvent choice and plant organ in determining antioxidant capacity and provides a meaningful reference within the Poaceae family. These findings provide a basis for further studies to evaluate the potential applications of *L. spartum* in pharmaceutical, nutraceutical, and food‐related fields.

### 3.3 Anticholinesterase Activity

Table 4 presents the AChE and BChE inhibitory activities of *L. spartum* L. extracts compared to galantamine.

**TABLE 4:** Anticholinesterase activity of the petroleum ether and methanol extracts of *L. spartum* L.

Samples	Extract	Anticholinesterase activity	
		AChE assay IC_50_ (µg/mL)	BChE assay IC_50_ (µg/mL)
Roots	PE MeOH	>200 *NA*	>200 >200
Leaves	PE MeOH	>200 >200	>200 >200
Galantamin		4.14 ± 0.25	40.39 ± 0.45

Abbreviations: NA, not active; PE, petroleum ether; MeOH, methanol.

The inhibitory activity of *L. spartum* L. extracts against AChE and BChE was generally weak. All tested extracts exhibited IC_50_ values higher than 200 µg/mL or were inactive (NA) against both enzymes, indicating very low or no inhibitory activity under the experimental conditions employed. In contrast, the reference inhibitor galantamine showed strong inhibitory activity, with IC_50_ values of 4.14 ± 0.25 µg/mL for AChE and 40.39 ± 0.45 µg/mL for BChE. Overall, these findings indicate that *L. spartum* extracts are not promising sources of cholinesterase inhibitors under the conditions evaluated.

Previous studies on members of the Poaceae family have reported variable anticholinesterase activities depending on the species, plant organ, extraction solvent, and phytochemical composition. For example, the essential oil of *Cymbopogon citratus* exhibited moderate AChE inhibitory activity with an IC_50_ of 2.86 ± 0.17 mg/mL [44], while the essential oil from fresh leaves of *Cymbopogon schoenanthus* showed stronger AChE inhibition with an IC_50_ of 0.26 ± 0.03 mg/mL [45]. Extracts of *Digitaria insularis* also demonstrated inhibitory activity, producing AChE inhibition rates of 34.8%, 43.2%, and 57.9% at 1 mg/mL for the hexane extract, ethyl acetate extract, and an active fraction, respectively [46]. Additionally, *C. citratus* essential oil exhibited IC_50_ values of 2.14 ± 0.18 µL/mL and 0.34 ± 0.07 µL/mL against AChE and BChE, respectively [47].

These variations in anticholinesterase activity among Poaceae species may be attributed to differences in phytochemical composition, particularly the presence of phenolic compounds, terpenoids, and other secondary metabolites known to contribute to enzyme inhibition.

### 3.4 Tyrosinase Inhibitory Activity

Table 5 shows that all tested extracts from *L. spartum*. exhibited no detectable tyrosinase inhibitory activity. This indicates that the extracts are inactive against this enzyme.

**TABLE 5:** Tyrosinase inhibitory activity of the Petroleum ether and methanol extracts of *L. spartum* L.

Sample	Extract	Tyrosinase inhibitory activity
		Tyrosinase assay IC_50_ (µg/mL)
Roots	PE MeOH	NA >200
Leaves	PE MeOH	NA 310.69
Kojic acid		12.55 ± 0.34

The tyrosinase inhibitory activity of *L. spartum* L. extracts was generally negligible. As shown in Table 5, most extracts exhibited no detectable inhibition, with IC_50_ values exceeding 200 µg/mL or being not active (NA). Only the methanolic leaf extract showed weak inhibition, with an IC_50_ of 310.69 µg/mL, which remains significantly higher than that of the standard inhibitor Kojic acid (IC_50_ = 12.55 ± 0.34 µg/mL). These results indicate that *L. spartum* L. extracts possess minimal or no tyrosinase inhibitory activity.

Comparable observations have been reported for other species within the Poaceae family. For example, fractions of *Spartina anglica* ethanol extracts (EtOH‐a and EtOH‐bg) at 100 µg/mL inhibited the tyrosinase‐mediated oxidation of L‐DOPA by 30.4% and 29.5%, respectively (Kim et al., 2022) [48]. Similarly, extracts of *Macrochloa tenacissima* (syn. *Stipa tenacissima*) showed measurable tyrosinase inhibition with an IC_50_ of 693.57 µg/mL [27]. These comparisons suggest that, although some Poaceae species can inhibit tyrosinase, the activity is generally weak, which aligns with the findings for *L. spartum* L

## 4 Limitations

The present study has some limitations that should be acknowledged. The biological activities were evaluated using crude extracts; therefore, the contribution of individual phenolic compounds to the observed antioxidant and enzyme inhibitory activities could not be established. In addition, the results were obtained exclusively from in vitro assays and should not be directly extrapolated to in vivo biological systems. Furthermore, the HPLC–DAD analysis was limited to the targeted phenolic standards included in the analytical method, and other bioactive constituents potentially contributing to the biological activities may not have been detected. Moreover, the biological assays were performed using technical replicates from a single extraction procedure; therefore, further studies involving independent extraction replicates are required to confirm the reproducibility and statistical robustness of the observed activities. Future studies should focus on the isolation and characterization of the major bioactive constituents, bioassay‐guided fractionation, evaluation of their individual and synergistic effects, and confirmation of their biological activities using appropriate in vivo models.

## 5 Conclusion

This study provides the first comprehensive characterization of the phenolic composition, antioxidant activity, and enzyme inhibitory properties of *L. spartum* L. extracts. HPLC–DAD analysis revealed an organ‐specific distribution of phenolic compounds, with the methanolic leaf extract characterized by relatively higher levels of ferulic acid, hesperidin, catechin, and apigenin than the root extract. Methanolic extracts, particularly those from leaves, exhibited moderate antioxidant activity, which may be related to their relatively higher phenolic content. In contrast, AChE, BChE, and tyrosinase inhibitory activities were generally weak, indicating limited potential of *L. spartum* as a source of cholinesterase or tyrosinase inhibitors under the experimental conditions evaluated. Overall, this study expands the current knowledge of the phytochemical composition and biological properties of *L. spartum* and provides a scientific basis for future studies aimed at the isolation and characterization of individual bioactive compounds, elucidation of their mechanisms of action, bioassay‐guided fractionation, and confirmation of their biological activities using appropriate in vivo models.

## Author Contributions

**Conceptualization**: Z.A. and M.Ö.; **Data curation**: D.Ç. and C.Ç.; **Formal analysis**: D.Ç. and C.Ç.; **Funding acquisition**: H.B., G.S.E.‐S., M.A.M.A., and W.E.; **Investigation**: R.A., D.Ç., and C.Ç.; **Methodology**: Z.A., D.Ç., and C.Ç.; **Project administration**: H.B., G.S.E.‐S., M.A.M.A., and W.E.; **Resources**: M.Ö.; **Supervision**: M.Ö. and S.G.; **Validation**: M.Ö.; **Visualization**: M.Ö.; **Writing – original draft**: R.A. and Z.A.; **Writing – review and editing**: H.B., G.S.E.‐S., M.A.M.A., W.E., and S.G. All authors have read and agreed to the published version of the manuscript .

## Use of Generative AI and AI‐Assisted Technologies in the Writing Process

The authors used an artificial intelligence (AI)‐assisted language tool solely to improve the language, grammar, clarity, and readability of the manuscript. The AI tool was not used for data generation, data analysis, interpretation of results, or scientific decision‐making. All scientific content was critically reviewed and verified by the authors, who take full responsibility for the accuracy and integrity of the manuscript.

## Ethics Statement

The conducted research is not related to either human or animal use, and the plant material was collected in Algeria from non‐protected natural habitats under an official collection permit issued by the Conservation of Forests (Direction de la Conservation des Forêts). The collection was carried out in accordance with the applicable national regulations governing scientific research and biodiversity conservation. The authors confirm that the study complies with the relevant national requirements concerning access to plant genetic resources and, where applicable, with the principles of the Nagoya Protocol.

## Conflicts of Interest

The authors declare no conflicts of interest.

## Data Availability

The data that support the findings of this study are available from the corresponding author upon reasonable request.
